# An online intervention for improving stroke survivors’ health-related quality of life: study protocol for a randomised controlled trial

**DOI:** 10.1186/s13063-019-3604-0

**Published:** 2019-08-09

**Authors:** Ashleigh Guillaumier, Sam McCrabb, Neil J. Spratt, Michael Pollack, Amanda L. Baker, Parker Magin, Alyna Turner, Christopher Oldmeadow, Clare Collins, Robin Callister, Chris Levi, Andrew Searles, Simon Deeming, Olivia Wynne, Alexandra M. J. Denham, Brigid Clancy, Billie Bonevski

**Affiliations:** 1 School of Medicine and Public Health, Faculty of Health and Medicine, The University of Newcastle, 1 University Drive, Callaghan, NSW 2308 Australia; 2 The University of Newcastle, School of Biomedical Sciences and Pharmacy, Faculty of Health and Medicine, 1 University Drive, Callaghan, NSW 2308 Australia; 3 Hunter Medical Research Institute (HMRI), New Lambton Heights, NSW 2305 Australia; 4 Hunter New England Local Health District, John Hunter Hospital, New Lambton Heights, NSW 2305 Australia; 5 IMPACT Strategic Research Centre, School of Medicine, Barwon Health, Deakin University, PO Box 291, Geelong, VIC Australia; 6 Department of Psychiatry, Level 1 North, Main Block, Royal Melbourne Hospital, University of Melbourne, Parkville, VIC Australia; 7 School of Health Sciences, Faculty of Health and Medicine, The University of Newcastle, 1 University Drive, Callaghan, NSW 2308 Australia

**Keywords:** Recurrent stroke, Prevention, Health behaviours, Health-related quality of life

## Abstract

**Background:**

Recurrent stroke is a major contributor to stroke-related disability and costs. Improving health-risk behaviours and mental health has the potential to significantly improve recovery, enhance health-related quality of life (HRQoL), independent living, and lower the risk of recurrent stroke. The primary aim will be to test the effectiveness of an online intervention to improve HRQoL among stroke survivors at 6 months’ follow-up. Programme effectiveness on four health behaviours, anxiety and depression, cost-effectiveness, and impact on other hospital admissions will also be assessed.

**Methods/design:**

An open-label randomised controlled trial is planned. A total of 530 adults will be recruited across one national and one regional stroke registry and block randomised to the intervention or minimal care control group. The intervention group will receive access to the online programme Prevent 2nd Stroke (P2S); the minimal care control group will receive an email with Internet addresses of generic health sites designed for the general population. The primary outcome, HRQoL, will be measured using the EuroQol-5D. A full analysis plan will compare between groups from baseline to follow-up.

**Discussion:**

A low-cost per user option to supplement current care, such as P2S, has the potential to increase HRQoL for stroke survivors, and reduce the risk of second stroke.

**Trial registration:**

Australian and New Zealand Clinical Trials Registry, ID: ACTRN12617001205325p. Registered on 17 August 2017.

**Electronic supplementary material:**

The online version of this article (10.1186/s13063-019-3604-0) contains supplementary material, which is available to authorized users.

## Background

Hypertension, smoking tobacco, low levels of physical activity, poor diet, and heavy levels of alcohol consumption are risk factors for recurrent stroke, [1] and can impact on health-related quality of life (HRQoL) [2–6]. Poor health behaviours can increase the risk of a second stroke due to their impact on each other, or being directly associated with the risk of a second stroke [7–12]. Further to these health-risk behaviours, higher rates of depression and anxiety have been found among stroke survivors and have been identified as barriers to stroke survivors seeking counselling, advice, and education to modify health-risk behaviours post stroke [13]. Depression and anxiety have also both been proposed to have a significant negative association with HRQoL [14–16].

Providing behavioural interventions, such as brief advice, education, and counselling to modify patient health-risk behaviours, is evidence-based best practice according to clinical guidelines for stroke management [17]. However, audits have found that only about half of all patients receive behavioural interventions at the time of discharge [18]. There are a number of reasons for this evidence-practice gap, including practitioner lack of training, confidence, and skills and time to provide counselling and advice [19].

An online intervention may be one way to address these barriers while meeting the APEASE criteria for designing scalable interventions of Affordability, Practicality, Effectiveness, and cost-effectiveness, Acceptability, Safety, and Equity [20]. In Australia, more than 86% of households report Internet access at home [21]. Surveys show that up to 80% of patients have an interest in supplementing clinician-delivered support with Internet-delivered information, [22] and reviews have consistently demonstrated the effectiveness of online interventions among comorbid populations for reducing pain, disability, depression, and anxiety [23–25].

### Aims

The primary aim of this study is to examine the effectiveness of an online healthy lifestyle intervention – Prevent 2nd Stroke (P2S) – at improving HRQoL among stroke survivors at 6 months’ follow-up. A secondary aim is to examine the cost-effectiveness of the intervention compared to usual care. Additional secondary aims are to examine the effect of the online P2S programme on:

1. Four health behaviours (smoking, alcohol use, fruit and vegetable intake, and moderate physical activity)
2. Mental health (depression and anxiety levels)
3. Self-reported physical functioning and independent living, and
4. Hospital admission for recurrent stroke, myocardial infarction and all other causes

## Methods/design

### Design

**Fig. 1:**
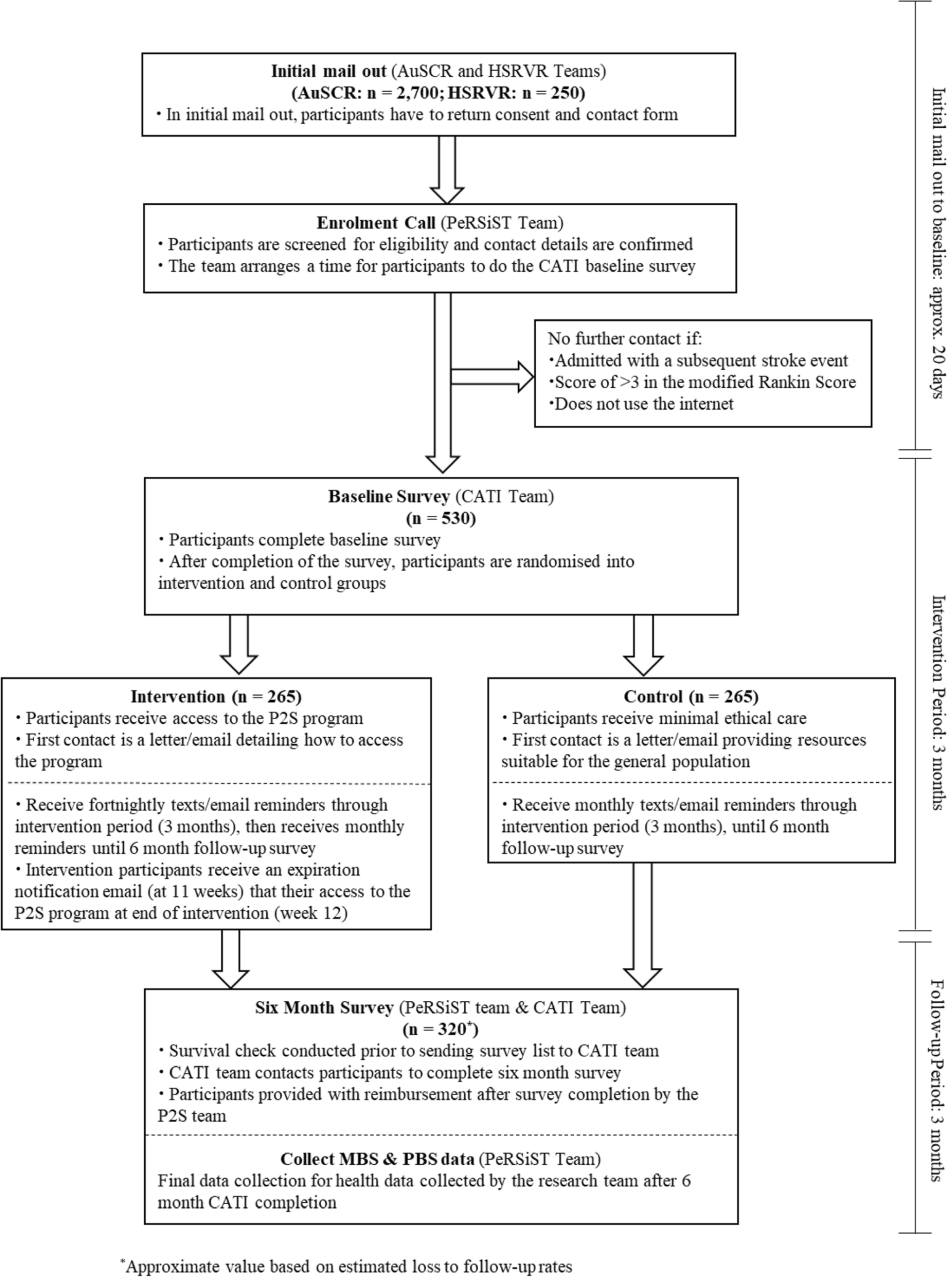
Schematic of the Prevent Second Stroke Trial (PeRSiST) study trial design

A prospective, open, blinded end-point, randomised controlled trial (PeRSiST trial) with stroke survivors as the unit of randomisation will be conducted. Outcome assessments will be blinded. A flow chart of the trial design appears in Fig. 1. The protocol has been written following the Standard Protocol Items: Recommendations for Interventional Trials (SPIRIT) advice (see Additional file 1).

### Setting

Participants will be recruited through two sources: (1) the Australian Stroke Clinical Registry (AuSCR) database, which is a national prospective opt-out enrolment registry for stroke and transient ischaemic attack (TIA) patients at 68 hospitals in six states of Australia, and (2) the Hunter Stroke Research Volunteer Register (HSRVR), this is a centralised database of people with stroke or TIA living in the Hunter region of the state of New South Wales who are willing to be contacted to participate in stroke research.

### Participants

#### Eligibility criteria

Individuals will be included if they: are aged 18 years and over; have been admitted to an AuSCR hospital for a first episode of care for acute stroke or TIA (indexed stroke event) *or* are registered with the HSRVR; are between 6 and 36 months post stroke; are sufficiently fluent in English; and have sufficient facility in Internet use via a home device (e.g. computer or tablet device) or are willing to use public Internet services (e.g. public library).

#### Exclusion criteria

Individuals will not be eligible to participate in the study if they have documented evidence of a previous stroke (second to the indexed stroke event) or experience disability at a level that may limit their use of the programme (determined using a score of ≤ 3 on the modified Rankin Scale) [26].

#### Withdrawal

If a participant wishes to withdraw consent, they will be able to at any point in time. They will be given the option to remove all their previously collected data or just remove consent for further data collection.

### Sample size

A sample of 160 individuals will be needed per treatment arm at follow-up to enable the detection of a 0.25 standard deviation difference (a 6-point difference) in HRQoL at 6 months with 80% power and 5% significance. This assumes a correlation between the baseline and follow-up HRQoL of 0.6, and a standard deviation of 24 points. Pilot data suggest that approximately 40% of participants recruited at baseline will complete follow-up at 6 months and, therefore, the research team aim to recruit 530 eligible consenting participants at baseline [27].

### Randomisation

A random number generator embedded in the computer-assisted telephone interview (CATI) software will be used to allocate study participants to either receive the intervention or standard care after all baseline questions have been answered. Individuals will be randomised at a ratio of 1:1 in permuted blocks of randomly varying size (e.g. four and six), stratified by state (New South Wales, Queensland, Western Australia, Victoria/Tasmania), and type of stroke (ischaemic, haemorrhagic, TIA, other). The statistician will be blinded to the treatment group.

### Recruitment and screening

AuSCR or HSRVR will screen individuals for eligibility criteria and will send out invitation packs on behalf of the trial research team to potentially eligible database registrants (i.e. individuals 6 to 36 months post first stoke, aged 18 years and older). Invitation packs will include a cover letter from AuSCR or HSRVR, a participant information statement describing the project Additional file 2 (Model participant information statement), and two consent forms (one for study participation Additional file 3 (Model participant consent form) and one to allow the researchers to access the individual’s health care data (Medicare and Pharmaceutical Benefits Schemes; MBS and PBS) from the national registry). Participants will be asked to sign and return the consent forms in a replied, paid envelope to the research team. Due to potential physical limitations in the population being studied, carers will be able to consent on behalf of individuals by signing the consent form and indicating that they are a carer. Upon receipt of the signed consent forms, potential participants will be telephoned by the research team (enrolment call) to be screened regarding Internet and email access, and disability using the brief nine-item modified Rankin Scale. Potential participants will be, given an opportunity to ask questions about the study. Upon eligibility confirmation, participants will complete a baseline CATI after which they will be randomised.

**Fig. 2:**
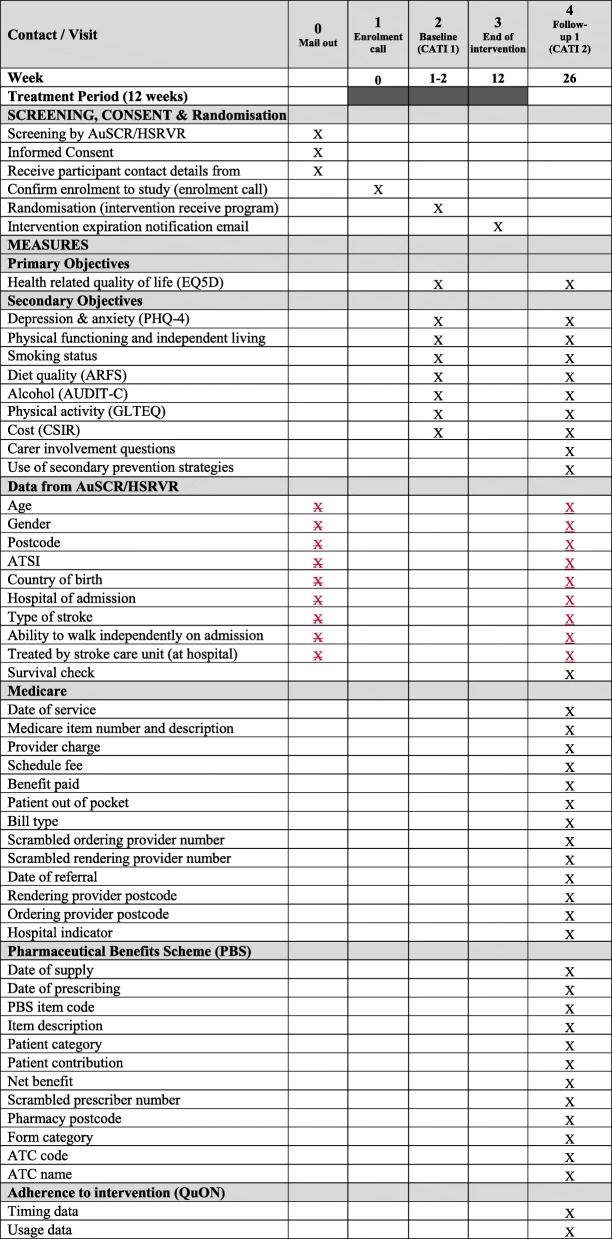
Assessment schedule of enrolment, interventions, and assessments for participants

Figure 1 outlines study recruitment and follow-up, while Fig. 2 contains an assessment schedule for the trial. To promote adherence to programme use and study follow-up, both groups will receive reminder texts (intervention fortnightly with additional email reminder; control monthly) during the intervention period of 12 weeks, then both groups will receive monthly reminder texts, including the approximate date for their next survey, until the 6-month follow-up. Intervention participants will have access to P2S for the 12 weeks. After 11 weeks, intervention participants will receive an email notifying them that their access to the P2S programme will expire within a week. Survival checks will be completed with AuSCR or HSRVR prior to telephone contact. All participants will be asked to complete a 6-month follow-up CATI. Participants will be reimbursed with an AU$50 gift card after the completion of the 6-month survey.

### Intervention

Participants randomised to receive the intervention will be sent an email and letter containing a link to P2S and their log-in details. The P2S programme is a modularised, tailored programme designed for individuals who have had a stroke to improve their HRQoL which may reduce their risk of a second stroke event. The P2S programme and its development is described in detail elsewhere [28]. Briefly, individuals are first presented with a loading module where they are asked questions to determine what information might be most relevant to the user. After completing the loading module, users are able to access the other seven modules (one module for each of the four health behaviours (Smoking, Alcohol Use, Diet, Physical Activity), one for Blood Pressure, one for Moods and Feeling, and the My Progress module (for participants to track their progress towards achieving goals).

During their 12-week access, intervention participants will be able to revisit sections of the programme as required, setting new goals, and recording progress throughout this time. Fortnightly text message and email reminders will be sent to prompt programme usage. Individuals will also receive a toll-free number that allows calls to the project team if they require assistance. Participants will be encouraged to use the online programme at least once a week and more often if they choose. Navigation through the programme will be flexible, with participants able to navigate around the programme freely, focussing on content areas of greatest interest or need.

### Minimal ethical care (control) group

Participants randomised to the control group will receive usual stroke follow-up care and will be sent an email and letter with links to Internet addresses of readily available, generic online health programmes designed for the general population (The Australian Department of Health ‘Quit Now’, ‘Australia’s Physical Activity and Sedentary Behaviour Guidelines’ and ‘Eat for Health’ sites; The National Health and Medical Research Council’s ‘Australian Guidelines to Reduce Health Risks from Drinking’; and the ‘MOODGym’ site). The use of an active control condition was chosen as it balances exposure to online health programmes and minimises the likelihood of changes in the treatment group due to non-specific therapeutic factors. P2S, which will only be accessible by entering user identification during the trial, will not be available to control group participants.

### Primary outcome measures

The primary outcome measure will be HRQoL, measured using the EuroQol 5 Dimensions, 5 Levels (EQ-5D-5 L) questionnaire [29], at 6 months. The EQ-5D-5 L consists of two stages of questions. Stage 1 contains five questions in the domains of mobility, self-care, usual activities, pain/discomfort, and mood (the EQ-5D). Each dimension has five levels: no problems, slight problems, moderate problems, severe problems, and extreme problems. The second stage adapts the EuroQol Visual Analogue Scale (EQ VAS), and asks respondents to numerically rate global health status from 0 to 100, and can be administered verbally [30, 31]. The justification for the EQ-5D-5 L is that it: (1) is very short and simple, taking 2–3 min and can be administered as a telephone interview; (2) is responsive to change in patients who have had a stroke; (3) is composed of multi-item scales and single items to examine differential effects; (4) has been used in, and is recommended for, stroke trials [32]; and (5) the construct, concurrent and discriminant validity, reliability, and acceptability (based on response rates and missing data) of the instrument with stroke patients has been well established [33].

### Secondary outcome measures

#### Cost-effectiveness

The main reportable outcomes for the cost-effectiveness analysis of the intervention will be (1) an average cost-effectiveness ratio, and (2) an incremental cost-effectiveness ratio, per Quality-adjusted Life Year (QALY). A modified version of the Client Service Receipt Inventory (CSRI) will be used in conjunction with the MBS and PBS data to determine use of health services (frequency, length and cost of physician consultations; allied health care; inpatient stays; outpatient episodes; medication use) and potentially other economic impacts (such as time off work due to disability) [34].

#### Smoking status

At baseline, a standard and previously validated item will assess smoking status ‘*Do you currently smoke any tobacco products?*’ (Daily, At least once a week, Less often than once a week, Not at all) [35]. At follow-up, 7-day point-prevalence abstinence (PPA) will be used to assess quit success using recommended items to determine the proportion of participants who have not smoked any tobacco in the preceding 7 days ‘*Have you smoked at least part of a cigarette in the last 7 days?*’ Seven-day PPA has high concurrent validity and reliability [36].

#### Alcohol consumption

The brief Alcohol Use Disorders Identification Test – Consumption (AUDIT-C), [37] will be used. The AUDIT-C is a three-item version of the more extensive AUDIT and has been found to be a valid and reliable screening tool for heavy drinking and alcohol abuse or dependence. Scoring for the AUDIT-C ranges from 0 to 12 with cut-offs of 3 for women (sensitivity 66–73%, specificity 91–94) [38, 39] and 4 for men are used to indicate heavy drinking (sensitivity 86%, specificity 72–89%) [39].

#### Fruit and vegetable intake

The Australian Recommended Food Score (ARFS) [40] will be used to assess fruit and vegetable consumption, a measure which has been validated in adults. The ARFS is strongly correlated with food frequency questionnaire nutrient intakes (fibre, vitamin A, beta-carotene, vitamin C, and minerals). To determine salt intake, participants will be asked ‘How often do you add salt to your cooking?’ and ‘How often do you use salt at the table?’ (Never/rarely, Sometimes, Usually, Always) [41].

#### Physical activity

Physical activity will be measured using the Godin Leisure Time Exercise Questionnaire (GLTEQ), [42] which measures the number of occurrences in a week in which a participant engages in periods of mild, moderate or vigorous exercise for 10 min or longer. Weights are then applied to the number of weekly occurrences of each type of exercise (mild, moderate, vigorous) and summed to calculate the number of metabolic equivalent or MET-minutes of exercise per week; it has acceptable reliability and validity.

#### Depression and anxiety

Depression and anxiety levels will be assessed using The Patient Health Questionnaire four-item (PHQ-4) [43]. The PHQ-4 is a validated and ultra-brief screening measure for both anxiety and depression. Scores range from 0 to 12, with categories of psychological distress being: none (0–2); mild (3–5); moderate (6–8); and severe (9–12). Subscale categories for anxiety and depression both range from 0 to 6 with a score of 3 or more considered positive.

#### Physical functioning and independent living

Physical functioning and independent living will be measured using the Barthel Index (BI) [44, 45] and Instrumental Activities of Daily Living (IADL) scales [46]. The BI measures activities of daily life on a 10-point scale related to self-care and mobility. The IADL questions attempt to capture the ability to live independently in the community.

#### Other secondary outcome measures

Other measures include hospital admission for recurrent stroke, myocardial infarction and all other causes, and data from medical health records. These data will be collected from national health care databases and will be used to inform resource-use assumptions in the cost-effectiveness analysis for patients utilising hospitals in health districts without access to an equivalent intervention.

### Additional measures

Patient and disease characteristics including age, gender, postcode, indigenous status, country of birth, hospital, type of stroke, whether the patient was able to walk independently on admission, and whether the patient was treated in a stroke care unit will also be collected. Adherence to the intervention, carer involvement, blood pressure awareness, and use of secondary prevention strategies will also be collected at 6 months. Programme usage data (i.e. timing and usage) will be collected as part of backend analytics from the web-platform hosting the P2S programme.

### Blinding

All baseline and follow-up telephone assessments will be conducted by independent health survey contractors blinded to experimental condition. Due to the nature of the interventions, blinding participant allocation and participants will be notified of the condition to which they have been assigned.

### Data collection

A commercial research company will be commissioned to collect baseline and follow-up data by CATI survey. MBS and PBS health data will be extracted from the national database at baseline and 6 months with the assistance of Department of Human Services, Canberra.

Study participant information and consent forms will have a unique study identification (ID) number. After formal enrolment into the trial participants will be assigned that unique study ID, allowing data to be stored in a re-identifiable format. The ID number will be used to identify participant data sets for comparison (i.e. baseline and follow-up surveys). Participant identifying details (i.e. names and contact details collected to enable follow-up) will be stored separate to other data, while the ID number will be attached to both.

### Statistical analysis

#### Primary analysis

Will be conducted according to the intention-to-treat (ITT) principle, comparing the change in HRQoL between groups from baseline to 6-month follow-up. The comparison will be done using a linear regression model, where the outcome variable will be the EQ-5D-5 L HRQoL score, and the main predictors of interest will be the baseline value of the outcome variable, and treatment group. The two stratifying variables, state of residence within Australia and stroke type at baseline, will be included in the model as covariates. The primary method of dealing with missing data will be through multiple imputation, this will provide a sensitivity analysis exploring how robust the results are when considering missing values. The missing data will be explored to test the underlying assumptions of multiple imputations. Adjusting for potentially imbalances in important prognostic variables and pattern mixture models will be used to correct for violations in the missing at random assumption.

#### Secondary analysis

Generalised linear mixed models will be used to examine between-group differences in changes in secondary outcomes between baseline and follow-up; appropriate distributional families and link functions will be used according to outcome type. The models will include fixed effects for time, group and the interaction between time and group, as well as stratification variables. Random subject-level intercepts will be included to account for serial correlations. Associations between programme usage and outcome will be examined where greater adherence to the programme will be expected to be associated with improved outcomes.

#### Cost-effectiveness analysis

In this study, a cost-utility analysis will be undertaken from the perspective of health care providers to identify whether the resources consumed to achieve the outcomes represent a cost-effective improvement over usual stroke follow-up care. The analysis will adhere to guidelines for conducting and reporting health economic evaluations [47, 48]. Given that the majority of resource savings are anticipated to arise following conclusion of the intervention, a patient lifetime time-horizon will be modelled founded on the trial outcomes. The primary measure of effectiveness for the economic evaluation will comprise HRQoL, as measured by EQ-5D-5 L, from which QALYs will be calculated using Australian utility weights. Resource utilisation for the respective trial arms will include: intervention development costs, health care costs derived from the AuSCR hospital *International Classification of Disease* (*ICD*) data, the CSRI data, and the MBS/PBS data. The *ICD* data will be translated to an estimate for cost differences between arms using allocations to diagnostic-related groups and according cost weights. It is implicitly assumed that differences between trial arms for non-stroke utilisation of inpatient services will be random. This assumption will be tested with the CSRI inpatient data and admitted patient data from a subset of health districts. Analysis of the QALYs and resource-use estimates will derive average and incremental cost-effectiveness ratios. Appropriate sensitivity and uncertainty analysis will be conducted.

A full statistical analysis plan will be written prior to analysis.

### Data monitoring

A data monitoring committee is not required for observational studies. In lieu of this committee, the research team will meet weekly to discuss the day-to-day running of the project and the full investigator team will be contacted monthly. This team will discuss any broader concerns arising from the research that cannot be managed by the research team, and will monitor the ethics and safety of the trial and will convene as required throughout the duration of the trial. If the research team is made aware of any negative events that may be the result of study participation, the research and investigator teams will be responsible for discussing the event, and any participation or protocol changes which may result, at their weekly/monthly meetings.

## Discussion

To date, there are limited findings on the impact of online behaviour-change interventions for improvements in HRQoL and the prevention of second stroke among stroke survivors. Determining if there is the potential to increase care provision through online platforms may help to bring care provision in line with current recommendations, or go further, and increase the quality of life of users and prevent further ill-health. The results of this randomised controlled trial should provide further evidence regarding online interventions and their ability to improve HRQoL and support stroke survivors.

### Trial status

Protocol version number and date: Version 3, dated 1 September 2018.

Recruitment start date: 1 March 2018.

Expected recruited completion date: 28 February 2019.

## Additional files

Additional file 1: Standard Protocol Items: Recommendations for Interventional Trials (SPIRIT) Checklist. (PDF 172 kb)

Additional file 2: Model participant information statement. Example information statement provided to potential participants about the trial. (PDF 177 kb)

Additional file 3: Model participant consent form. Example consent form provided to potential participants to enrol in the trial. (PDF 222 kb)

## Data Availability

Not applicable.

## Abbreviations

APEASE: Affordability, Practicality, Effectiveness, and cost-effectiveness, Acceptability, Safety, and Equity
AuSCR: Australian Stroke Clinical Registry
CATI: Computer-assisted telephone interview
CSRI: Client Service Receipt Inventory
HRQoL: Health-related quality of life
HSRVR: Hunter Stroke Research Volunteer Register
*ICD*: *International Classification of Disease*
ID: Identification
ITT: Intention-to-treat
MBS: Medicare Benefits Scheme
P2S: Prevent 2nd Stroke
PBS: Pharmaceutical Benefit Scheme
PeRSiST: Prevent Second Stroke Trial
QALY: Quality-adjusted Life Year
TIA: Transient ischaemic attack
