# Supplementary material for: An online intervention for improving stroke survivors’ health-related quality of life: study protocol for a randomised controlled trial
Source: Trials. 2019 Aug 9;20:491. doi: 10.1186/s13063-019-3604-0 (PMC6688335; doi:10.1186/s13063-019-3604-0)
Supplement: Supplementary file 2 — Model participant information statement. Example information statement provided to potential participants about the trial. (PDF 177 kb) [file 13063_2019_3604_MOESM2_ESM.pdf]

Principal Investigator Name X
Department X
University X
Address X
Town X
abcd@efjhijklm.edu.au

THE UNIVERSITY OF NEWCASTLE AUSTRALIA

Information Statement for the Research Project

Healthy Living After Stroke: An online intervention for improving stroke survivor health behaviours and quality of life.

You are invited to take part in the research project identified above, conducted by a team of researchers led by Prof. [Principal Investigator] from the School of Medicine and Public Health at the University of Newcastle. This study is funded by the National Health and Medical Research Council (NHMRC).

Why is the research being done?

Many lifestyle behaviours are linked to stroke and Transient Ischemic Attack (TIA). These include smoking, risky alcohol drinking, poor diet, and lack of exercise. We have developed an online program called “Prevent 2nd Stroke” to help stroke and TIA survivors avoid having another stroke or TIA.

This study will test whether the “Prevent 2nd Stroke” program works and has a lasting impact on the lives of stroke and TIA survivors who use it. To do this, we will conduct a randomised trial. This means, if you agree to participate, you have a 50/50 chance of receiving the online program. If you don’t receive the online program, you will still receive usual care. Everybody who takes part in the trial will be asked to complete two telephone surveys. We will then compare those who used the program to those who did not in order to tell whether the “Prevent 2nd Stroke” online program is effective at improving health and cost-effective.

Who can take part in the research?

You can take part if you:

- Are aged 18 years and over
- Were admitted to an Australian Stroke Clinical Registry (AuSCR) associated hospital for acute stroke or TIA
- It has been between 6 and 36 months since your acute stroke or TIA
- understand English
- Have access to the internet at home (e.g., computer or tablet device) or at public internet services (e.g., public library, internet cafe)

What choice do you have?

Participation in this research is entirely your choice. Only those people who give their informed consent will be included in the project. Whether or not you decide to participate, your decision will not disadvantage you in any way, or be disclosed to anyone outside of the research team. If you do decide to participate, you may withdraw from the project at any time without giving a reason and you always have the option of withdrawing any data which identifies you.

What would you be asked to do?

If you agree to take part, please return your completed consent form in the reply-paid envelope provided.

After we receive your consent form, a member of the research team will call you to answer any questions you may have about the project and if you agree, enrol you in the study. During this phone call, we will also arrange a convenient time for you to complete the first of two telephone surveys that occur over the 6-month study period. We may follow up with email or letter if we are unable to contact you via telephone. After you have completed the first survey, we will notify you (via letter and email) whether you have been randomly allocated to receive the “Prevent 2nd Stroke” program (intervention group) or not (control group).

- *Intervention group*: the notification letter and e-mail will contain the details of the Prevent 2nd Stroke website and your personal program log-in details. You will have access to the online program for 12 weeks. During these 12 weeks we will send you fortnightly text messages and emails to remind you to use the online program. The program contains tailored healthy lifestyle information. You can work through the program at your own pace, setting goals, monitoring progress and focussing on the content areas of most interest to you. The program contains six modules on 1) blood pressure; 2) smoking; 3) alcohol; 4) activity; 5) nutrition, and; 6) feelings and mood.

- *Control group*: the notification letter and e-mail will contain a list of websites that provide information about lifestyle factors. You may look at these resources at your convenience.

Participants in both groups will complete one telephone survey 6 months after the first survey. All participants will receive a monthly text message reminder about the date of their next telephone survey. The surveys ask about you, your stroke or TIA, your health and, in the follow-up surveys, your use of any online health programs. If you are unable to complete the surveys yourself, you are able to nominate a friend or family member to complete the surveys on your behalf.

We would also like to consent to access your data that is held in the Australian Stroke Clinical Registry (AuSCR). This data would include: a) information about you like age, gender, postcode, country of birth; b) stroke-related information like clinical and hospital data for your first stroke/TIA, and c) your Medicare number. ***You are providing consent for the research team to collect this data from AuSCR by signing the study consent form.*** We are asking to collect this information from the AuSCR to reduce the amount of questions we ask you in the surveys. We would also like to use your Medicare number only to check if you have been admitted to hospital again.

You will also be asked to fill out a consent form authorising the study access to your complete Medicare and Pharmaceutical Benefits Scheme (PBS) data as outlined on the back of the consent form. Medicare collects information on your doctor visits and the other costs. The PBS collects information on the prescription medicines you have filled at pharmacies. The consent form is sent securely to the Department of Human Services who holds this information confidentially. This information will be used to determine costs during the study period:

- *Medicare data*: we will use data from Medicare to identify costs relating to your stroke/TIA care (e.g. medicines, doctors appointments, use of health services) during the study period. ***We will ask you to complete a form providing your consent to release the Medicare and PBS claims information to our study.*** The consent form contains an example of the type of information that would be given to the study.

How much time will it take?

Each survey will take around 30 minutes to do. At the end of the second survey you will be given a $50 gift card for the time and costs of doing the survey for us.

The first telephone survey will take about 30 minutes to complete. The second survey, 6 months later, will take around 30 minutes to do.

If you receive the Prevent 2nd Stroke program, you may use it at your own pace over 12 weeks. In total it will take about 1-2 hours to work through the complete program, however you may wish to revisit sections which are more relevant to you. If you are in the control group, you will receive a list of websites where you can find more health information that you can use at your leisure.

What are the risks and benefits of participating?

We cannot promise you any specific benefit from taking part in this research. But, we hope that the online program gives stroke and TIA survivors a better understanding of how to be healthy and avoid having a second stroke or TIA.

We do not think there are any risks to participating in this project. However, if completing the surveys brings up any personal issues you would like to discuss, the following free telephone services may be helpful: StrokeLine on 1800 787 653 or Lifeline on 13 11 14. Alternatively, you should contact your usual doctor.

How will your privacy be protected?

All the information collected from you for the study will be treated confidentially, and only authorised members of the research team will have access to it. All paperwork will be stored in a locked filing cabinet in the lead researcher's rooms at the University of Newcastle. All electronic information will be stored in password protected files on a secure, University-hosted online platform, with access available only to authorised research team members. At the end of the study, all information excluding the Medicare and PBS data will be stored for a minimum of 7 years at the University of Newcastle, after which time all paper documents will be shredded and all electronic information permanently deleted. Medicare and PBS data will be stored for only 7 years before being destroyed.

How will the information collected be used?

The information collected may be published in scientific journals and be presented at relevant scientific conferences. Individual participants will not be identified in any reports arising from the project. A summary of the results of the study will available at the study's conclusion. Information collected in this study may contribute towards the completion of a student thesis.

What do you need to do to participate?

Please read this Information Statement and be sure you understand its contents before you consent to participate. If there is anything you do not understand, or you have questions, please contact the research team.

If you would like to participate, please complete and return the attached consent form in the reply paid envelope provided. This will be taken as your informed consent to participate.

Research team

[Principal Investigator]<br>University X	
**Investigator name X**<br>Affiliation	**Investigator name X**<br>Affiliation
**Investigator name X**<br>Affiliation	**Investigator name X**<br>Affiliation
**Investigator name X**<br>Affiliation	**Investigator name X**<br>Affiliation
**Investigator name X**<br>Affiliation	**Investigator name X**<br>University of Newcastle
**Investigator name X**<br>Affiliation	**Investigator name X**<br>Affiliation
**Investigator name X**<br>Affiliation	**Investigator name X**<br>Affiliation
**Investigator name X**<br>Affiliation	**Investigator name X**<br>Affiliation

Further information

If you would like further information please contact the research team on 1800 316 788, or e-mail the team at prevent2ndstroke@newcastle.edu.au

Thank you for considering this invitation.

Professor [Principal Investigator]

Complaints about this research

This project has been approved by the University's Human Research Ethics Committee, Approval No. H-2017-0051.
Should you have concerns about your rights as a participant in this research, or you have a complaint about the manner in which the research is conducted, it may be given to the researcher, or, if an independent person is preferred, to the Human Research Ethics Officer, Research Services, NIER Precinct, The University of Newcastle, University Drive, Callaghan NSW 2308, Australia, telephone (02) 4921 6333, email Human-Ethics@newcastle.edu.au.
