# Supplementary material for: An online intervention for improving stroke survivors’ health-related quality of life: study protocol for a randomised controlled trial
Source: Trials. 2019 Aug 9;20:491. doi: 10.1186/s13063-019-3604-0 (PMC6688335; doi:10.1186/s13063-019-3604-0)
Supplement: Supplementary file 3 — Model participant consent form. Example consent form provided to potential participants to enrol in the trial. (PDF 222 kb) [file 13063_2019_3604_MOESM3_ESM.pdf]

Principal Investigator Name X
Department X
University X
T: xx xxxx xxxx
F: xx xxxx xxxx
e: abcd@efghijklm.edu.au

Consent Form

Healthy Living After Stroke: An online intervention for improving stroke survivor health behaviours and quality of life.

I agree to participate in the above research project and give my consent freely.

I understand that the project will be conducted as described in the Information Statement, a copy of which I have retained.

I have had all questions answered to my satisfaction.

I understand I can withdraw from the project at any time and do not have to give any reason for withdrawing.

I agree to:

- Being randomised to either receiving access to the online Prevent 2nd Stroke program (intervention group) or not (control group)
- Complete two telephone surveys over the next 6 months.
- Receive telephone calls, text, and email reminders as part of my study participation.
- Be contacted via by e-mail or letter if I am unable to be contacted by phone
- Allow the research team to collect my de-identified, stroke-related and hospital admissions data through the Australian Stroke Clinical Registry (AuSCR) database during the study period.
- Allow the research team to seek additional specific written consent (via supplementary consent forms) to collect administrative information regarding the costs associated to my stroke/TIA from Medicare, the Pharmaceutical Benefits Scheme (PBS), and hospital medical files.

Please sign and date the form below as well as completing your contact details over the page. Return this form to the research team with the prepaid envelope provided

Signature: ____

Date: _________________

If you would **not** like to participate, and **would not like to be contacted any further**, please tick this box ☐

Principal Investigator Name X
Department X
University X
T: xx xxxx xxxx
F: xx xxxx xxxx
e: abcd@efghijklm.edu.au

Your Contact Details

As you are aware, we will need to contact you upon receiving these details and again in 6 months' time to complete study surveys with you.

So that we can contact you, we would like you to give us your contact details.

Name: ____

Postal address: ____

____

Home phone: _______________________ Mobile: __________________________

Email: ____

Best time for researcher to call: ____

Would you like a summary of the results of this study to be sent to you when the project is finished? **Yes / No** *(please circle one)*

Nominated Carer/Friend/Family Member

I consent to assist in the survey as a proxy, and help the participant to participate in the study.

Name: ____

Signature: _______________________________________

Date: ________________

This project has been approved by the University of Newcastle Human Research Ethics Committee [H-2017-0051].
